# Comprehensive evaluation of artifact reduction and tissue recovery effects of metal artifact reduction technique based on full-reference metric

**DOI:** 10.1038/s41598-023-38516-9

**Published:** 2023-07-19

**Authors:** Yunsub Jung, You Seon Song, In Sook Lee, Seung Joon Rhee

**Affiliations:** 1 Center for Mathematical Modeling of Knee Osteoarthritis, Department of Materials and Production, Aalborg University, Åalborg, Denmark; 2 Department of Radiology, Biomedical Research Institute, Pusan National University Hospital, Busan, Republic of Korea; 3 School of medicine, Pusan National University, Busan, Republic of Korea; 4 Department of Orthopedic Surgery, Biomedical Research Institute, Pusan National University Hospital, Busan, Republic of Korea

**Keywords:** X-ray tomography, Computational biophysics, Biomedical engineering

## Abstract

For the comprehensive evaluation of metal artifact reduction (MAR) technique, not only the removal of metal artifacts but also the evaluation of the area restored by MAR is required. We propose a method to comprehensively evaluate the effect by MAR in this study. We have conducted the computed tomography scan to acquire both the evaluation image and the reference image for the full-reference based evaluation. The evaluation image and reference image were reconstructed into 24 image sets according to the tube potentials, image reconstruction method, and use of the MAR technique. Images of two different positions were selected according to the distance from metal and material (bone, tissue) distribution, and bone and tissue were automatically segmented in both evaluation and reference images. The values of full width at half the maximum (FWHM) and centroid were extracted after Gaussian modeling of each segmented region. Then, we computed four evaluation metrics (FWHM_NM_: non-MAR to non-metal ratio of FWHM, FWHM_M_: MAR to non-metal ratio of FWHM, CENT_NM_: non-MAR to non-metal ratio of centroid, CENT_M_: MAR to non-metal ratio of centroid), and the MAR image and non-MAR image were compared. The overlap ratio automatically segmented from the evaluation image and reference image were position 1 (bone: 99.61%, tissue: 99.23%) with 80 kVp, position 1 (bone: 99.32%, tissue: 99.56%) with 120 kVp, position 2 (bone: 99.20%, tissue: 99.73%) with 80 kVp, and position 2 (bone: 99.23%, tissue: 99.67%) with 120 kVp. The FWHM_NM_ showing the change of image pixel value by metal artifact was calculated as (bone: 1.32–1.46, tissue: 1.08–1.16) at 80 kVp and (bone: 1.19–1.27, tissue: 1.02–1.05) at 120 kVp. More metal artifacts occurred at 80 kVp tube potential. Regardless of the tube potential and image reconstruction method, the MAR showed an overall artifact reduction effect (1 < FWHM_M_ < FWHM_NM_). However, distortion of pixel values occurred due to the MAR in regions where metal artifacts were high in proximity to metal (1 < FWHM_NM_ < FWHM_M_). Overall, the average value of the medium was maintained (CENT_M_: 0.98–1.03) after MAR application, but there was a change of image value in region around the metal (CENT_M_: 0.97–1.11). In this study, we propose a new method to evaluate the effect of metal artifacts and MAR technique using full-reference based method. Metal artifacts, effect of MAR technique, and side-effect caused by MAR technique were quantitatively analyzed through proposed method. There are some limitations in applying it to clinical imaging since our method is a reference-based evaluation. However, our experimental results were important for understanding the effects of the MAR technique and its functional properties.

## Introduction

Metallic implants cause various artifacts during computed tomographic (CT) scans. These metal artifacts are an obstacle for diagnosis because they impair the image quality of adjacent tissues of the metal material^1^. Photon starvation and beam hardening that occur when x-rays pass through metal are major causes of metal artifacts. These effects cause artifacts such as bright and dark streak artifacts and dark fields in CT images^2,3^. In order to remove the causes of metal artifacts such as photon starvation and beam hardening, there is a method by using a hardware filtration and by changing the CT scan parameters (higher peak voltage, spatial resolution, and temporal resolution)^4^. However, these methods have many limitations and ultimately do not prevent the occurrence of metal artifacts.

A lot of metal artifact reduction (MAR) techniques have been developed and commercialized to remove metal artifacts shown in CT image using post-processing methods^5–8^. Most of MAR techniques commonly find metal and metal artifacts region, then use a method of recovering the damaged surrounding area due to the artifact. Since this is a sort of approximation and the sinoram affects the whole image reconstruction, there is a possibility of compromising the original information of the tissue. Therefore, some MAR techniques use methods that only remove areas of severe artifacts and retain weak artifacts to minimize damage to the contaminated tissue^6,7^.

Since the MAR effect is very different depending on the CT image quality and the MAR technique, many studies have been conducted to evaluate the MAR effect^9–16^. Existing studies have mainly evaluated MAR performance under various conditions (CT scan protocol, various MAR products, image reconstruction method) using a commercial phantom^9,10^ simulating a human body, using a customized phantom^13–15^ to evaluate MAR performance according to the environment of metal and surrounding materials, or MAR in clinical images^11–13,16^. For MAR evaluation, a qualitative evaluation^11–16^ was performed by radiologists using the score system, or a quantitative evaluation was performed by measuring the mean and standard deviation based on the Hounsfield unit (HU) value, structural similarity index measure (SSIM), mean square error (MSE) and edge reduction ratio^9–16^.

Most of the existing research evaluating the MAR technique are studies evaluating only the effect of removing metal artifacts by the MAR technique. Artifacts caused by the MAR technique have been reported. These new artifacts have been observed in the form of “focal linear”, “nodular dark density lesions”^16^, or “pseudocemented appearance”^12^, and these artifacts can potentially lead to misdiagnosis^16^. Therefore, it is very important to check the image pixel value change caused by the MAR in the restoration process by the MAR technique, and a comprehensive evaluation of the MAR is required.

Previously published methods and metrics for quantitative evaluation of MAR technique has several limitations. First, the metrics that calculate the SSIM and MSE between the reference and evaluation image cannot evaluate artifacts by MAR. Since the metal artifact area removed by MAR has a relatively larger change in pixel value than the artifact area by MAR, these metrics only lead to the result that the MAR image is closer to the reference image than the non-MAR image. Also, these metrics must be calculated at the same pixel location of both images (reference, evaluation). However, since the reference and evaluation image are obtained by different CT scans, even if the phantom is fixed to the CT table, a slight change in position occurs due to moving of CT table and vibration caused by the rotation of the gantry. Therefore, new evaluation method and metric are needed to evaluate the effect of MAR and its side effects. In this study, we provide a methodology to separately evaluate the effect of MAR and its side effects through changes in the distribution of pixels in the medium.

The image quality evaluation includes the no-reference method that does not use a reference image and full-reference method that use a reference image for evaluation^17^. For comprehensive evaluation of the effect of artifact removed by the MAR technique and recovery of the normal tissue contaminated by the metal artifact, it is necessary to compare with the reference image (without metal insertion) scanned in the same position as the evaluation image (with metal insertion)^18^. The full-reference method using a reference image is suitable for evaluation using a phantom, however, it has a disadvantage that cannot be applied to real clinical practice. The purpose of this study is to present a method to comprehensively evaluate the effectiveness of MAR technique. For this, the experiment was designed in consideration of the tissue recovery performance evaluation after the MAR technique along with the artifact reduction effect by the MAR technique.

## Materials and methods

### Data acquisition

**Figure 1:**
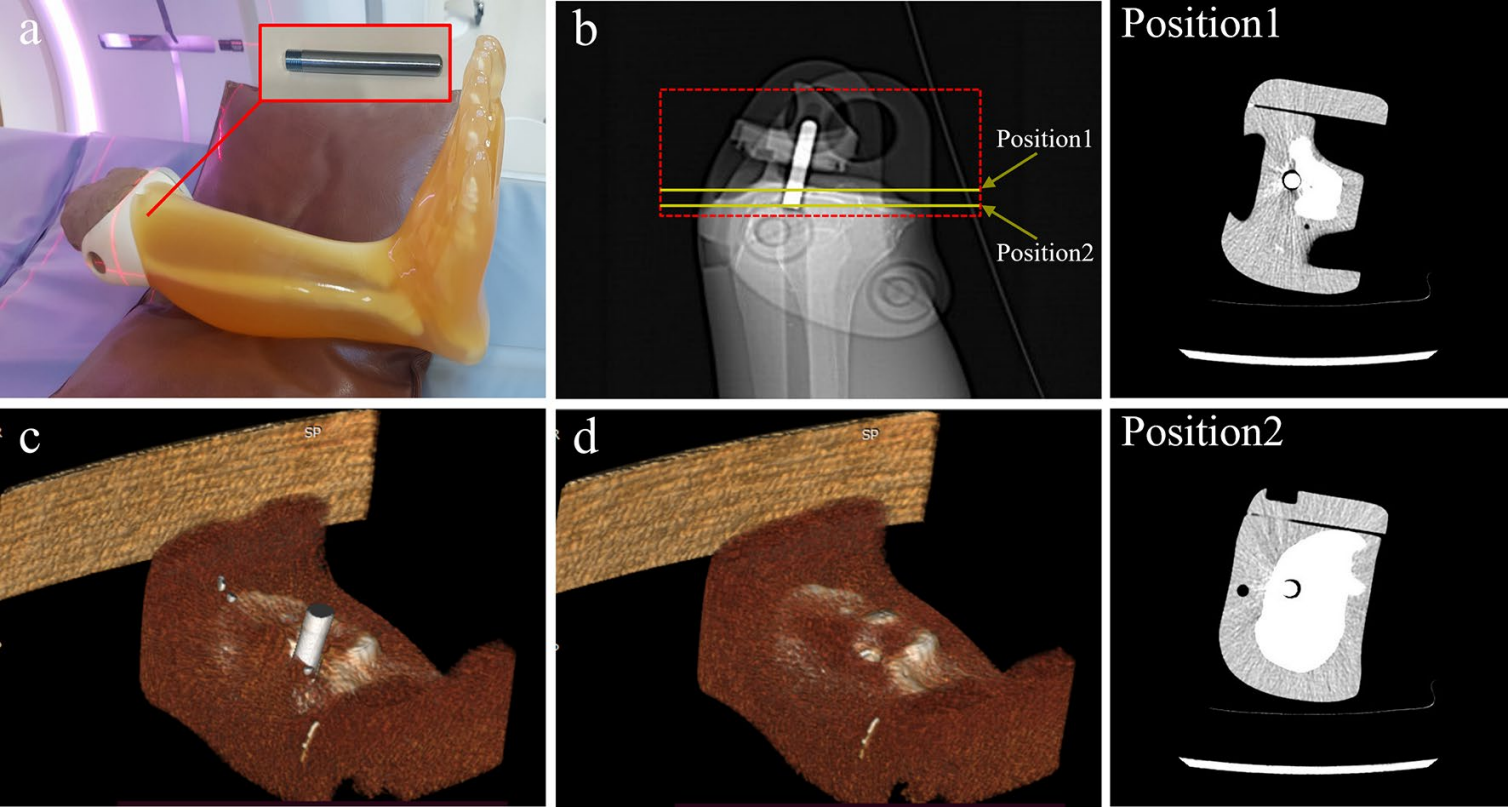
Phantom CT scan of the lower extremity including the tibia. For evaluation and reference images, phantoms with and without metal were scanned at the same location. (**a**) Stainless steel metal with a diameter 8 mm inserted into proximal tibia, (**b**) Scout image of scan area. Analysis images were manually extracted from two positions according to the distribution of bones and tissues around the metal, (**c**) 3D rendering image of phantom with metal, (**d**) 3D rendering image of phantom without metal.

**Figure 2:**
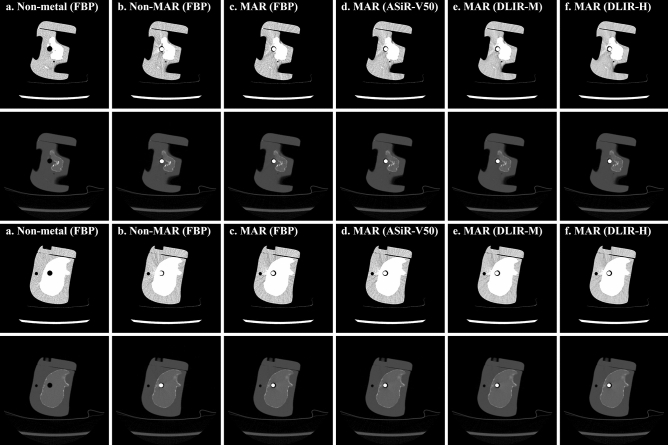
Images of two locations for analysis. These images are scanned at a tube potential of 80 kVp and a tube current of 50 mA (1.06 mGy), where images in rows 1 and 3 are window width/level (400/40) and rows 2 and 4 are images of window width/level (4024/1035). Each row shows (**a**) non-metal (FBP), (**b**) non-MAR (FBP), (**c**) MAR (FBP), (**d**) MAR (ASiR-V50), (**e**) MAR (DLIR-M) and (**f**) MAR (DLIR-H) images in order.

CT (Revolution Apex, GE Healthcare, USA) scan was performed using a phantom (PBU-60, Kyoto Kagaku, Japan) that mimics the low extremity part including tibia and fibula (Fig. 1, Table 1). After CT scanning by inserting a metal (stainless steel) with a diameter 8 mm into the proximal tibia, the metal was removed from phantom then, CT scan was performed again. To accurately match the phantom positions in both evaluation and reference images, all scans were performed using axial mode without moving the CT table. Human intervention is required in the process of inserting and removing a metal, and at this time, the experiment was conducted so that the position of the phantom does not change. The metal-removed scan data is used as a reference image for full-reference evaluation. Since the position of the phantom in the evaluation image (scan 1,3) and the reference image (scan 2,4) must be exactly the same, the insertion and removal of metal was performed very precisely so that the phantom did not move on the CT table. A total of 4 scans were performed according to tube potential (80 and 120 kVp) and metal insertion, and each scan was reconstructed into a total of 24 sets of images according to four image reconstruction methods (GE Healthcare, USA) and MAR technique (Smart metal artifact reduction, GE Healthcare, USA) application (Table 1). The four image reconstruction methods used are filtered back-projection (FBP), adaptive statistical iterative reconstruction (ASiR-V50), and two levels of deep learning-based image reconstruction (DLIR-Medium and High). The numbers following ASiR-V represent the blending ratio between ASiR-V and FBP (i.e., ASiR-V30 = ASiR-V * 0.3 + FBP * 0.7). DLIR was based on deep neural network (DNN) provides three selectable reconstruction levels (Low, Medium, High) depending on the strength of the noise reduction. The manufacturer currently provides only the standard kernel in DLIR, so all reconstructions used the standard kernel. Images of two positions with different distributions of bone and tissue around the metal were selected for analysis (Fig. 2). The position1 is an image in which metal is inserted in the tissue area, and there is a partial portion of tibia around the metal, and position2 is an image with metal inserted into the proximal tibia and a tissue on the outside of the tibia. Images from these two positions were manually selected from the same axial position from 24 data sets. A total of 48 images were used for analysis, all of which were axial images, with slice thickness (0.625 mm), matrix size (512 × 512 pixels), field of view (200 × 200 mm) and pixel size (0.39 × 0.39 mm).

**Table 1:** Experiment conditions and CT data acquisition.

Scan^a^	Tube potential (kVp)	Tube current (mA)	CTDI_vol_ (mGy)	Metal insertion	MAR^b^	Image reconstruction			
						FBP	ASiR-V50	DLIR-M	DLIR-H
1	80	50	1.06	O	O	O	O	O	O
					X	O	O	O	O
2	80	50	1.06	X	X	O	O	O	O
3	120	50	3.15	O	O	O	O	O	O
					X	O	O	O	O
4	120	50	3.15	X	X	O	O	O	O

*CTDI*_*vol*_ computerized tomography dose index volume, *MAR* metal artifact reduction, *FBP* filtered back-projection, *ASiR-V* adaptive statistical iterative reconstruction veo, *DLIR* deep learning-based image reconstruction (Medium, High).

^a^Scans 1 and 3 were reconstructed with and without MAR applied, respectively. Scans 2 and 4 are experiments in which no metal is inserted, which are scanned for reference image.

^b^The MAR technique is performed together during the image reconstruction process.

### Rigid registration

**Figure 3:**
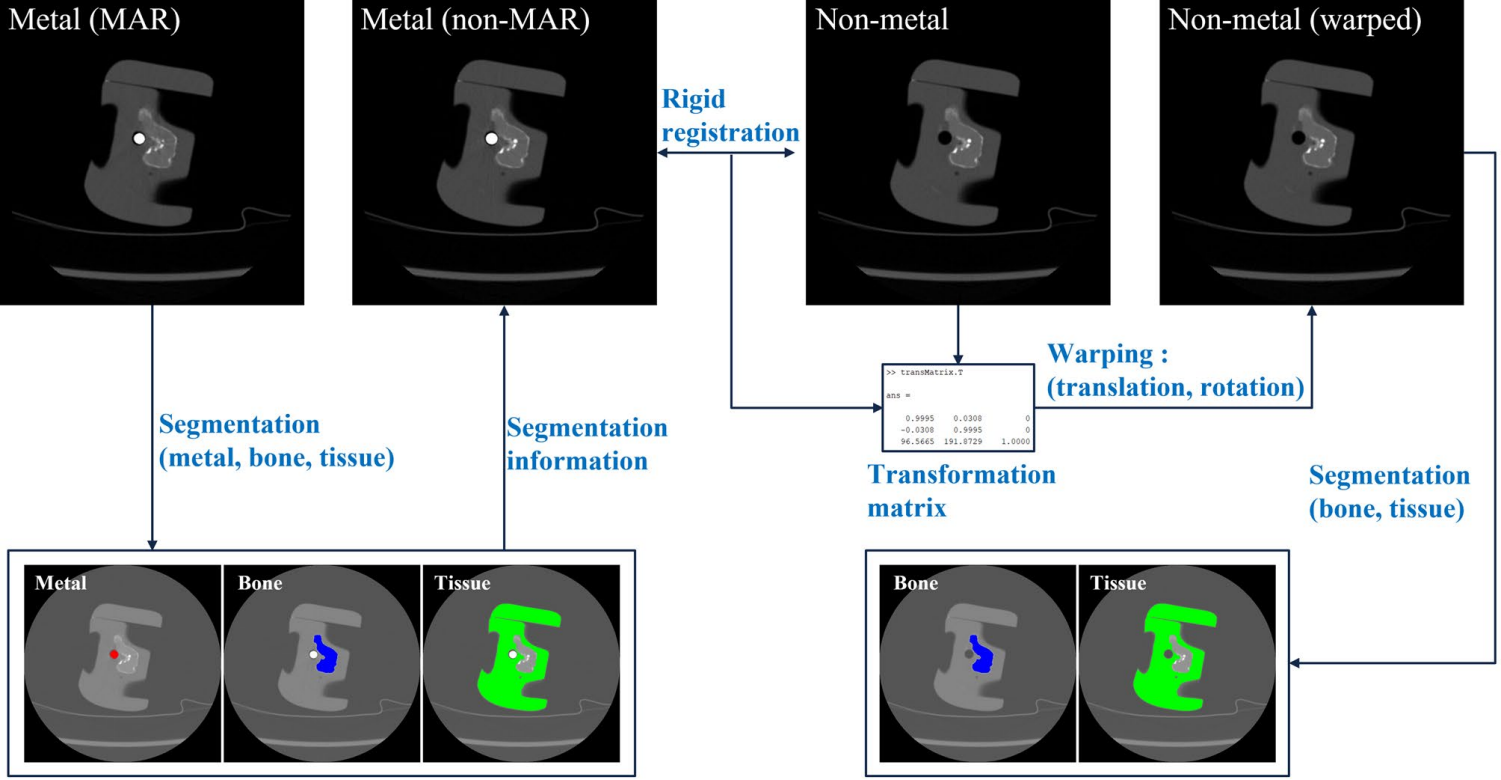
Schematic diagram of the pre-processing for analysis region segmentation. The non-MAR image is registered with non-metal image. Metal, bone, and tissue are segmented from the MAR image, and bone and tissue are segmented in the non-metal image after registration. Registration and segmentation are all performed automatically by an algorithm without human intervention.

MAR image and non-MAR image are evaluated based on a non-metal image (reference image). For this purpose, the position of the evaluation structure in the three images must be exactly the same. Since the MAR image and the non-MAR image are derived from the same CT scan, the location of the phantoms within the image are exactly the same. However, since the non-metal image is a different CT scan with the two evaluation images (MAR and non-MAR image), the location of the phantom within the image is not always the same. Although the CT scan was performed by minimizing the movement of the phantom, image registration was performed to compensate for a very small position change caused by vibration of the CT gantry and movement of the table. Non-metal image (moving image) was registered based on non-MAR images (target image) using rigid registration (Fig. 3). Rigid registration keeps the same shape and size after a rigid transformation because it is a registration that only performs translation and rotation^19^. The pixel value was slightly changed by interpolation in transformation process, but this change was very small and did not show a big difference from the original image.

### Structures segmentation using gradient vector flow

In this study, the quantitative evaluation of MAR uses the pixel distribution characteristics of structures inside the phantom. For this, three structures were segmented automatically (Fig. 3). First, metal was extracted in MAR image with metal. Threshold method and label size filtering were used for metal segmentation. The metal in the center of the phantom is a cylindrical structure with a very high intensity HU, and since we know the information about its actual diameter and cross-sectional area, other labels remaining as result of the threshold method are removed as noise. Here, we used a threshold (metal > 2000 HU) and a label size filter (noise label < 100 pixel). Second, the Gradient Vector Flow (GVF) model, one of active contour model (ACM) techniques^20^, was used to segment bone and tissue structures. The ACM is a technique that calculates information about a deformable model or edge line in an image using internal and external energy functions. GVF is defined as a vector field *vf*(*x*,*y*) = [*u*(*x*,*y*), *v*(*x*,*y*)] and computes the edge line of an object by minimizing the energy function (Eq. 1)^21–23^.

1 $$E_{GVF} = \int \int \mu \left( {u_{x}^{2} + u_{y}^{2} + v_{x}^{2} + v_{x}^{2} } \right) + \left| {\nabla f} \right|^{2} \left| {vf - \nabla f} \right|^{2} dxdy$$

where μ is the regularization parameter that is a positive value. $f\left( {x, y} \right) = \left| {\nabla G_{\sigma } \left( {x, y} \right)*I\left( {x, y} \right)} \right|$ means the edge map of image, $\nabla G_{\sigma } \left( {x, y} \right)$ is a 2D Gaussian function with standard deviation (σ) and gradient operator ($\nabla$). Also, the $I\left( {x, y} \right)$ represents the pixel value of image, where x and y are the horizontal and vertical positions of the image, respectively. Finally, the *E*_*GVF*_ is calculated by transforming it into the Euler–Lagrange equation (Eq. 2–3). In each bone and tissue edge image obtained by the GVF model, the inside of each structure was filled using the flood fill method.

2 $$\frac{\partial u}{{\partial t}} = \mu \nabla^{2} u - \left| {\nabla f} \right|^{2} \left( {u - f_{x} } \right)$$

3 $$\frac{\partial v}{{\partial t}} = \mu \nabla^{2} v - \left| {\nabla f} \right|^{2} \left( {v - f_{y} } \right)$$

The metal, bone, and tissue were segmented in MAR image containing metal. And the bones and tissues were segmented in warped non-metal image after registration (Fig. 3). Since the MAR and the non-MAR image are obtained from the same CT scan, the positions of the internal structures are exactly the same. Therefore, segmentation information from MAR image was used in non-MAR image. As a result, the phantom position in the three images is the same since the metal and the non-metal image were registered. Segmentation was performed using the DLIR-M image to match the analysis region among all image reconstruction, and this segmentation information was equally used in other image reconstructions.

### Anatomical modeling based on pixel distribution

**Figure 4:**
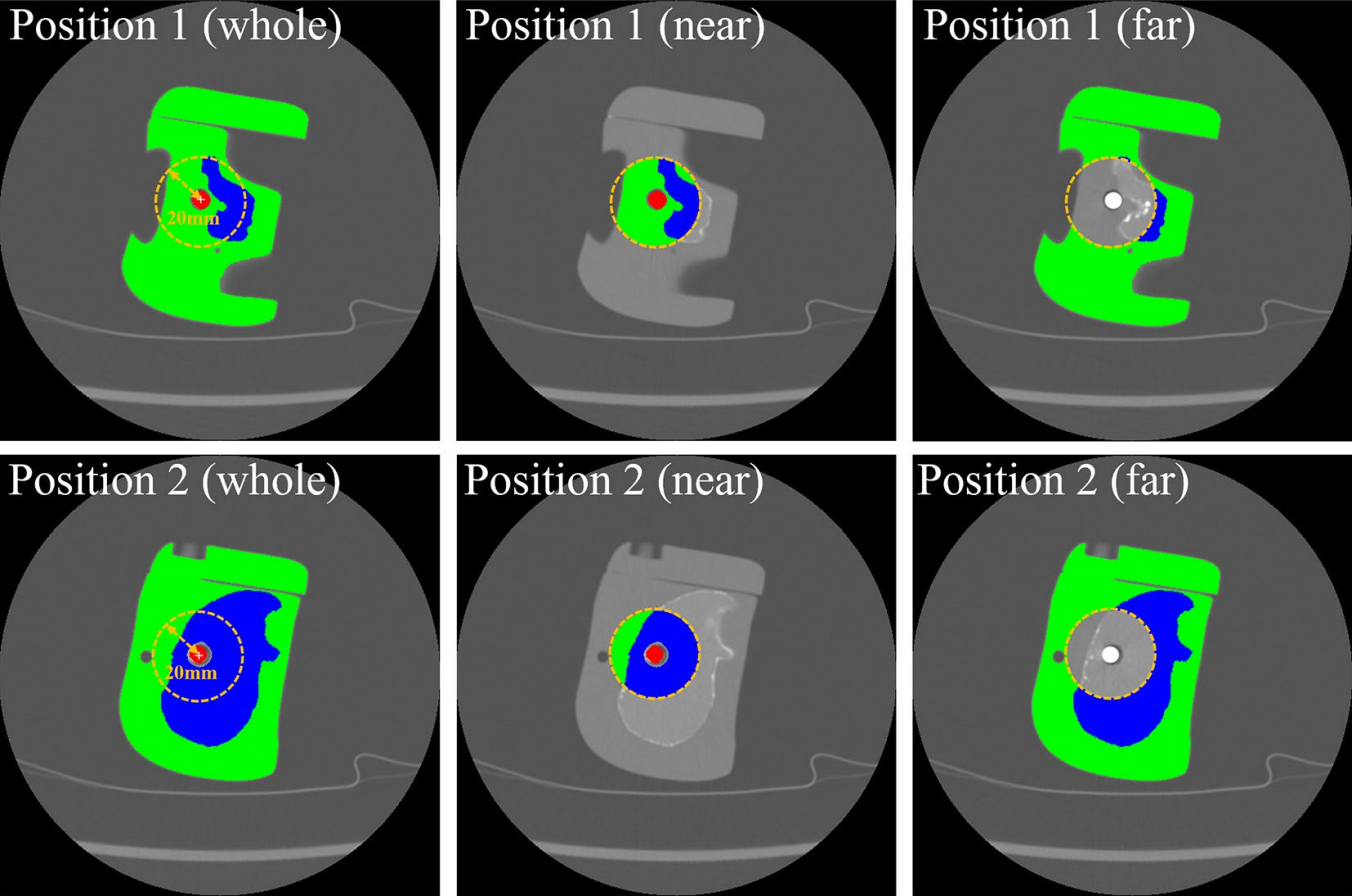
Division of the analysis region. The segmented labels of the bone and tissue are divided into a near region and a far region according to the distance (20 mm) from the center of the metal.

In order to evaluate the characteristics of MAR according to the distance based on the metal causing metal artifacts, we divided the segmented structures into two areas. Each segmented bone and tissue are separated into near and far regions, respectively, based on 20 mm from the center of the metal (Fig. 4). The 20 mm distance, which was the criterion for region separation, was determined by two musculoskeletal radiologists based on the effect of metal artifacts on the phantom image.

**Figure 5:**
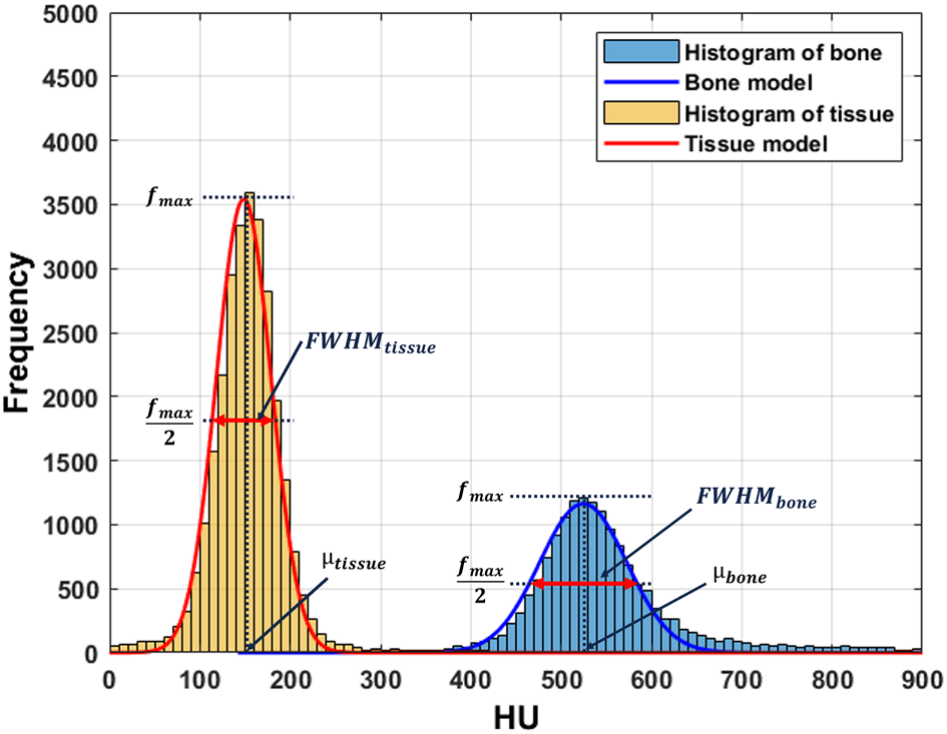
Anatomical modeling of bone and tissue regions. Bone and tissue regions were modeled using Gaussian curves in the distribution histogram of bones and tissues automatically segmented from the image. FWHM represents the x-direction cross-sectional length of the Gaussian curve at the half point (*f*_*max*_/2) of the y-axis peak (*f*_*max*_) of the model, and μ represents the x value at the *f*_*max*_ point. The x-axis of the graph represents the HU value, and the y-axis represents the frequency of the histogram.

To evaluate comprehensively the effect of MAR and side effects by MAR technique, we modeled the pixel distribution of bone and tissue regions in evaluation images (MAR and non-MAR image) and reference images (non-metal image), respectively (Fig. 5). First, after obtaining a histogram using pixel distribution in each region of already segmented bone and tissue, the distributions of each structure were modeled as a Gaussian curve (Eq. 4).

4 $$f\left( x \right) = \frac{1}{{\sqrt {2\pi \sigma^{2} } }}exp\left[ { - \frac{{\left( {x - \mu } \right)^{2} }}{{2\sigma^{2} }}} \right]{ }$$

The full width at half the maximum (FWHM) and centroid of model are extracted from the bone and tissue model modeled with the Gaussian curve, respectively (Fig. 5). Here, the centroid (*μ*_*bone*_ and *μ*_*tissue*_) is mean and $\sigma$ is a standard deviation of the Gaussian equation, and FWHM (*FWHM*_*bone*_ and *FWHM*_*tissue*_) becomes the horizontal length of the curve at the half point (*f*_*max*_/2) of the y-maximum (*f*_*max*_) of the model. The *FWHM*_*bone*_ and *FWHM*_*tissue*_ is calculated using the σ values of the bone and tissue model (Eq. 5).

5 $$FWHM_{bone\, or\, tissue} = { }\sqrt {2{\text{In}}2} {\upsigma }_{bone\, or \,tissue}$$

Here, the centroid represents the average HU value of each segmented structure, and the FWHM represents the pixel distribution of the segmented area as a single numerical value. If the pixel value inside the structure is changed by metal artifact or MAR technique, the width of the model will change, and the FWHM value will represent this.

### Quantitative evaluation metrics

Using the centroid and FWHM extracted for the overall reference-based evaluation, four evaluation metrics (*FWHM*_*NM*_ : non-MAR to non-metal ratio of FWHM, *FWHM*_*M*_ : MAR to non-metal ratio of FWHM, *CENT*_*NM*_ : non-MAR to non-metal ratio of centroid, *CENT*_*M*_ : MAR to non-metal ratio of centroid) were calculated as follows (Eqs. 6–7). Here, the reference image becomes non-metal image, and the evaluation image becomes MAR image and non-MAR images. If the value of FWHM_NM_ is greater than FWHM_M_ (FWHM_NM_ > FWHM_M_), it indicates that metal artifacts are well removed by MAR. In the opposite case (FWHM_NM_ < FWHM_M_), it means that the original image is distorted due to the side effect of MAR.

6 $$\left\{ {\begin{array}{*{20}c} {FWHM_{NM} = FWHM \,of \,non\hbox{-}MAR / FWHM \,of \,non\hbox{-}metal} \\ {FWHM_{M} = FWHM\, of\, MAR / FWHM\, of \,non\hbox{-}metal } \\ \end{array} } \right.$$

7 $$\left\{ {\begin{array}{*{20}c} {CENT_{NM} = centroid \,of \,non\hbox{-}MAR / centroid\, of \,non\hbox{-}metal} \\ {CENT_{M} = centroid\, of \,MAR / centroid\, of \,non\hbox{-}metal } \\ \end{array} } \right.$$

### Data analysis

The quantitative evaluation method including image registration and segmentation algorithm proposed in this study was implemented using software development tool (MATLAB, 2019a, MathWorks, Natick, USA). A one-way ANOVA test was performed to compare FWHM, centroid, *FWHM*_*NM*_, *FWHM*_*M*_, *CENT*_*NM*_, and *CENT*_*M*_ under each condition (image reconstruction method, analysis region, tube potential). In addition, evaluations among the specific conditions were compared using Bonferroni analysis. All statistical analyses were performed using statistical software (SPSS, version 22.0, IBM Corp., Armonk, NY, USA), and *P* values less than 0.05 were considered statistically significant.

## Results

### Results of Structure Segmentation

Table 2 shows the results of automatic segmentation of bone and tissue. Each bone and tissue segmented is separated into near and far regions according to the distance from the metal center. Overlap ratio between evaluation region and reference region was calculated to confirm the agreement of the analysis region (Eq. 8). The overlap ratio showed that the mean and standard deviation were 99.82 ± 0.15 in position1 image with 80 kVp, 99.56 ± 0.55 in position1 image with 120 kVp, 99.60 ± 0.40 in position2 image with 80 kVp, and 99.70 ± 0.32 in position2 image with 120 kVp, respectively, indicating that the two evaluation areas were performed in almost the same area.

8 $${\text{Overlap ratio }} = { }\frac{{\left( {{\text{Evaluation}}_{{{\text{area}}}} \cap {\text{Reference}}_{{{\text{area}}}} } \right)}}{{\left( {{\text{Evaluation}}_{{{\text{area}}}} \cup {\text{Reference}}_{{{\text{area}}}} } \right)}} * 100$$

**Table 2:** Segmentation results.

Region	Object	Position1 (80 kVp, 50 mA)			Position1 (120 kVp, 50 mA)		
		Evaluation image^a^	Reference image^b^	Overlap ratio^c^	Evaluation image	Reference image	Overlap ratio
	Metal	344	–	–	348	–	–
Whole	Bone	3354	3370	99.61	3335	3315	99.32
	Tissue	29,737	29,746	99.23	29,670	29,650	99.56
Near	Bone	2416	2424	99.70	2410	2400	99.14
	Tissue	4854	4850	99.31	4849	4839	98.54
Far	Bone	938	946	99.71	925	915	99.64
	Tissue	24,883	24,896	99.65	24,821	24,811	99.57

Region	Object	Position2 (80 kVp, 50 mA)			Position2 (120 kVp, 50 mA)		
		Evaluation image^a^	Reference image^b^	Overlap ratio^c^	Evaluation image	Reference image	Overlap ratio
	Metal	314	–	–	314	–	–
Whole	Bone	16,306	16,351	99.20	16,326	16,341	99.23
	Tissue	28,572	28,613	99.73	28,672	28,623	99.67
Near	Bone	5901	5940	99.38	5934	5940	99.37
	Tissue	1618	1642	99.59	1650	1642	99.52
Far	Bone	10,405	10,411	98.81	10,392	10,401	99.15
	Tissue	26,954	26,971	99.56	27,022	26,981	99.25

Metal, bone, and tissue were segmented using MAR images for evaluation, and bone and tissue were segmented using non-metal image for reference. All segmentation was performed on each DLIR-M image, and all values in table except overlap ratio represent the number of pixels in the segmented region.

^a^Images with metal, scan1 (80 kVp) and Scan3 (120 kVp) data set from Table 1.

^b^Images without metal, Scan2 (80 kVp) and Scan4 (120 kVp) data set from Table 1.

^c^Overlap ratio = (evaluation area ∩ reference area)/(evaluation area ∪ reference area) * 100.

### Quantitative evaluation according to metal insertion

**Figure 6:**
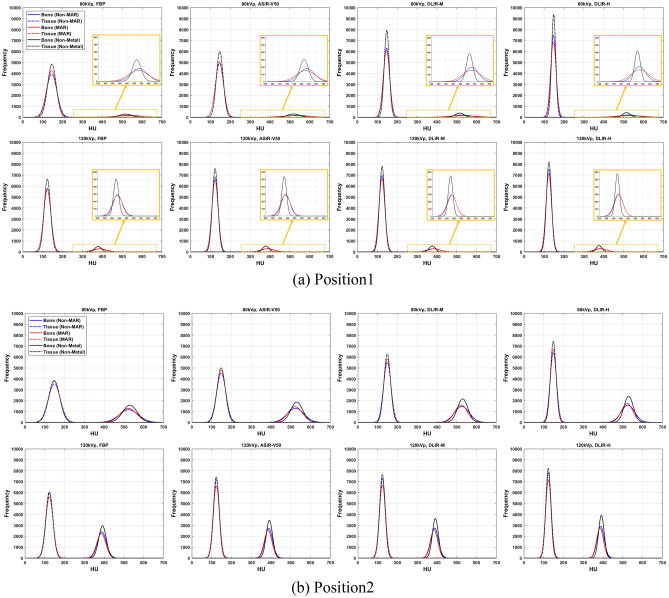
Modeling results of bone and tissue according to X-ray dose (1.06 mGy for 80 kVp, 50 mA and 3.15 mGy for 120 kVp, 50 mA), image reconstruction method (FBP, ASiR-V50, DLIR-M, and DLIR-H), and metal insertion.

Figure 6 shows the modeling results of bone and tissue according to X-ray dose, image reconstruction method, application of MAR, and metal insertion. FWHM and centroid of bone and tissue were extracted (Supplementary Fig. 1 and 2, Supplementary Table 1 and 2) from the model of all regions (whole, near, and far). The larger the FWHM value, the wider the range of pixel values inside a specific structure. The centroid represents the average HU value inside the segmented area. Under the same conditions (position, region, tube potential, and anatomic structures) for non-MAR, MAR, and non-metal images, the FWHM value decreased in the order of FBP > ASiR-V50 > DLIR-M > DLIR-H (all, *p* < 0.01; in Supplementary Table 1^1^. It was confirmed that the FWHM decreased as the tube potential (120 kVp > 80 kVp) increased, and thus it was less affected by the artifacts caused by the metal at a high applied tube potential (all, *p* < 0.01; in Supplementary Table 1). The near region close to the metal showed a significant increase in FHWM compared to the whole and far regions, confirming that the effect of the metal was severe (all, *p* < 0.01; in Supplementary Table 1). Overall, the centroid values of non-MAR and MAR image were similar compared to the non-metal image (approximately 0–3%, *p* < 0.05). However, the centroid values showed significant difference of 7–11% (80 kVp, non-MAR), 9–11% (80 kVp, MAR), 5–6% (120 kVp, non-MAR), and 8–10% (120 kVp, MAR) in the near tissue region of position 2 image (all, *p* < 0.01; in Table 4).

### Comparison of MAR and non-MAR images based on full-reference metrics

Tables 3 and 4 shows the results using the reference-based metrics *FWHM*_*NM*_, *FWHM*_*M*_, *CENT*_*NM*_, and *CENT*_*M*_ calculated using FWHM and centroid. Overall, the *FWHM*_*M*_ was smaller than *FWHM*_*NM*_ (non-MAR of FWHM > MAR of FWHM ≥ non-metal of FWHM, all, *p* < 0.05), so after reduction the metal artifact, the distribution of pixel values was maintained close to the non-metal image (Table 3), but *FWHM*_*M*_ was larger than *FWHM*_*NM*_ for some structures in the near area, which is close to the metal. This phenomenon was greatest in all bone regions (whole, near, and far) at 80 kVp of position 1, and the near tissue region at 80 kVp and 120 kVp of position 2. In particular, in position 1, where most of the bones are distributed in the near region around the metal, *FWHM*_*M*_ > *FWHM*_*NM*_ in all regions (whole, near, and far). However, it showed *FWHM*_*M*_ < *FWHM*_*NM*_ in the 120 kvp condition of position 1. Values of the *CENT*_*NM*_ and *CENT*_*M*_ did not show a significant difference in all experimental sections (0–4%, all, *p* < 0.01). Though the centroid values of non-MAR and MAR images were significantly different from non-metal image in the near region of position 2, CNM and CM showed a difference of up to 4% (0–4%).

**Table 3:** Non-MAR to non-metal ratio of FWHM (*FWHM*_*NM*_)^a^ and MAR to non-metal ratio of FWHM (*FWHM*_*M*_)^b^.

Region		Position1 (80 kVp, 50 mA, Bone)				Position1 (120 kVp, 50 mA, Bone)			
		FBP	ASiR-V50	DLIR-M	DLIR-H	FBP	ASiR-V50	DLIR-M	DLIR-H
Whole	$FWHM_{NM}$	1.81	1.82	2.08	2.22	1.95	2.02	2.04	2.09
	$FWHM_{M}$	2.18	2.23	2.85	3.12	1.75	1.83	1.95	2.02
Near	$FWHM_{NM}$	1.82	1.83	2.12	2.33	2.25	2.31	2.38	2.46
	$FWHM_{M}$	2.46	2.50	3.21	3.58	2.11	2.19	2.33	2.42
Far	$FWHM_{NM}$	1.67	1.70	1.80	1.80	1.31	1.38	1.38	1.41
	$FWHM_{M}$	1.71	1.77	2.23	2.34	1.18	1.25	1.37	1.41

Region		Position1 (80 kVp, 50 mA, Tissue)				Position1 (120 kVp, 50 mA, Tissue)			
		FBP	ASiR-V50	DLIR-M	DLIR-H	FBP	ASiR-V50	DLIR-M	DLIR-H
Whole	$FWHM_{NM}$	1.22	1.20	1.21	1.20	1.13	1.10	1.09	1.07
	$FWHM_{M}$	1.13	1.18	1.28	1.35	1.15	1.14	1.14	1.13
Near	$FWHM_{NM}$	1.65	1.72	2.02	2.22	1.49	1.45	1.46	1.48
	$FWHM_{M}$	1.17	1.23	1.58	1.76	1.43	1.48	1.48	1.52
Far	$FWHM_{NM}$	1.19	1.18	1.20	1.20	1.12	1.09	1.09	1.07
	$FWHM_{M}$	1.13	1.19	1.29	1.37	1.14	1.13	1.14	1.13

Region		Position2 (80 kVp, 50 mA, Bone)				Position2 (120 kVp, 50 mA, Bone)			
		FBP	ASiR-V50	DLIR-M	DLIR-H	FBP	ASiR-V50	DLIR-M	DLIR-H
Whole	$FWHM_{NM}$	1.32	1.38	1.42	1.46	1.19	1.20	1.25	1.27
	$FWHM_{M}$	1.16	1.18	1.23	1.25	1.19	1.19	1.21	1.22
Near	$FWHM_{NM}$	1.83	2.04	2.04	2.14	1.58	1.71	1.84	1.89
	$FWHM_{M}$	1.67	1.85	2.02	2.16	1.97	2.18	2.32	2.43
Far	$FWHM_{NM}$	1.14	1.17	1.22	1.24	1.04	1.03	1.06	1.09
	$FWHM_{M}$	0.99	1.00	1.04	1.06	1.01	1.01	1.05	1.06

Region		Position2 (80 kVp, 50 mA, Tissue)				Position2 (120 kVp, 50 mA, Tissue)			
		FBP	ASiR-V50	DLIR-M	DLIR-H	FBP	ASiR-V50	DLIR-M	DLIR-H
Whole	$FWHM_{NM}$	1.08	1.11	1.14	1.16	1.02	1.03	1.05	1.05
	$FWHM_{M}$	1.00	1.03	1.05	1.08	1.07	1.11	1.13	1.14
Near	$FWHM_{NM}$	1.46	1.68	1.73	1.83	1.42	1.63	1.55	1.62
	$FWHM_{M}$	1.67	2.00	2.40	2.76	2.11	2.67	2.67	2.93
Far	$FWHM_{NM}$	1.07	1.09	1.12	1.14	1.00	1.01	1.03	1.04
	$FWHM_{M}$	0.98	1.01	1.03	1.06	1.05	1.07	1.10	1.10

*FWHM* full width at half the maximum, *FBP* filtered back-projection, *ASiR-V* adaptive statistical iterative reconstruction veo, *DLIR* deep learning-based image reconstruction (Medium, High).

^a^$FWHM_{NM}$ = FWHM of non-MAR/FWHM of non-metal image.

^b^$FWHM_{M}$ = FWHM of MAR/FWHM of non-metal image.

**Table 4:** Non-MAR to non-metal ratio of centroid (*CENT*_*NM*_)^a^ and MAR to non-metal ratio of centroid (*CENT*_*M*_)^b^.

Region		Position1 (80 kVp, 50 mA, Bone)				Position1 (120 kVp, 50 mA, Bone)			
		FBP	ASiR-V50	DLIR-M	DLIR-H	FBP	ASiR-V50	DLIR-M	DLIR-H
Whole	$CENT_{NM}$	1.03	1.03	1.03	1.03	1.02	1.02	1.02	1.02
	$CENT_{M}$	1.02	1.02	1.03	1.03	1.03	1.03	1.03	1.03
Near	$CENT_{NM}$	1.04	1.04	1.04	1.03	1.03	1.03	1.03	1.03
	$CENT_{M}$	1.02	1.02	1.04	1.04	1.03	1.03	1.03	1.03
Far	$CENT_{NM}$	1.01	1.01	1.01	1.01	1.02	1.02	1.01	1.01
	$CENT_{M}$	1.02	1.02	1.02	1.02	1.03	1.03	1.02	1.02

Region		Position1 (80 kVp, 50 mA, Tissue)				Position1 (120 kVp, 50 mA, Tissue)			
		FBP	ASiR-V50	DLIR-M	DLIR-H	FBP	ASiR-V50	DLIR-M	DLIR-H
Whole	$CENT_{NM}$	0.99	0.99	0.99	0.99	1.00	1.00	1.00	1.00
	$CENT_{M}$	0.99	0.99	0.99	0.99	1.00	1.00	0.99	0.99
Near	$CENT_{NM}$	1.00	1.00	1.00	1.00	1.02	1.02	1.02	1.02
	$CENT_{M}$	1.00	1.01	1.01	1.01	1.02	1.02	1.02	1.02
Far	$CENT_{NM}$	0.99	0.99	0.99	0.99	0.99	0.99	0.99	0.99
	$CENT_{M}$	0.99	0.99	0.99	0.99	0.99	0.99	0.99	0.99

Region		Position2 (80 kVp, 50 mA, Bone)				Position2 (120 kVp, 50 mA, Bone)			
		FBP	ASiR-V50	DLIR-M	DLIR-H	FBP	ASiR-V50	DLIR-M	DLIR-H
Whole	$CENT_{NM}$	0.99	0.99	0.99	0.99	0.99	0.99	0.99	0.99
	$CENT_{M}$	0.99	0.98	0.99	0.99	0.98	0.98	0.98	0.98
Near	$CENT_{NM}$	0.98	0.98	0.99	0.99	0.99	0.99	0.99	0.99
	$CENT_{M}$	0.97	0.97	0.97	0.98	0.97	0.97	0.97	0.97
Far	$CENT_{NM}$	0.99	0.99	1.00	1.00	0.99	0.99	0.99	0.99
	$CENT_{M}$	0.99	0.99	0.99	0.99	0.99	0.99	0.99	0.99

Region		Position2 (80 kVp, 50 mA, Tissue)				Position2 (120 kVp, 50 mA, Tissue)			
		FBP	ASiR-V50	DLIR-M	DLIR-H	FBP	ASiR-V50	DLIR-M	DLIR-H
Whole	$CENT_{NM}$	1.00	1.00	1.00	1.00	1.01	1.01	1.01	1.01
	$CENT_{M}$	1.00	1.00	1.00	1.00	1.00	1.00	1.00	1.00
Near	$CENT_{NM}$	1.10	1.11	1.08	1.07	1.06	1.06	1.05	1.05
	$CENT_{M}$	1.10	1.11	1.10	1.09	1.10	1.09	1.09	1.08
Far	$CENT_{NM}$	1.00	1.00	1.00	1.00	1.00	1.01	1.01	1.01
	$CENT$	1.00	1.00	1.00	1.00	1.00	1.00	1.00	1.00

*FBP* filtered back-projection, *ASiR-V* adaptive statistical iterative reconstruction veo, *DLIR* deep learning-based image reconstruction (Medium, High).

^a^$CENT_{NM}$ = centroid of non-MAR/centroid of non-metal image.

^b^$CENT_{M}$ = centroid of MAR/centroid of non-metal image.

## Discussion

**Figure 7:**
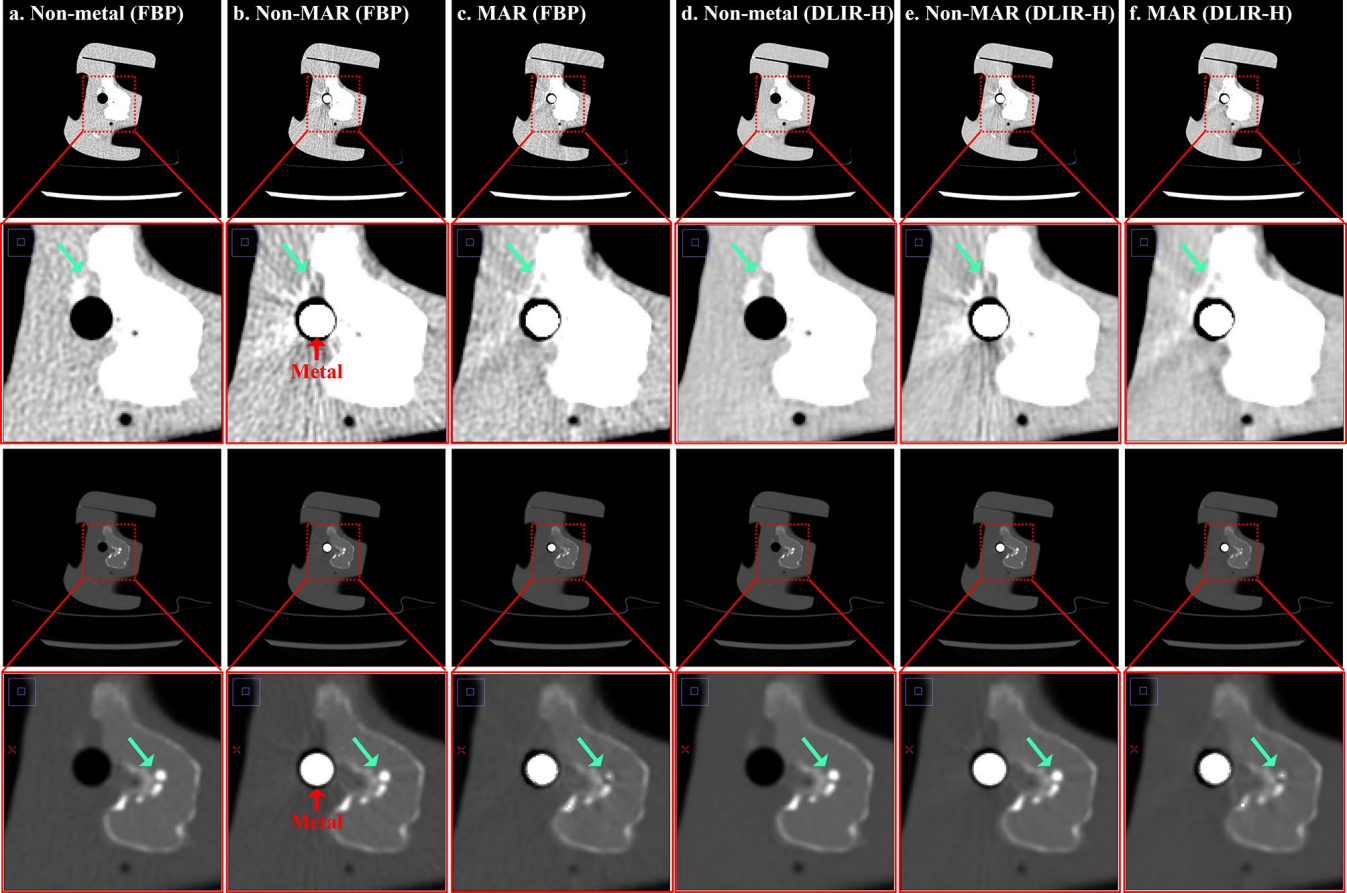
This figure shows the metal artifacts and the results after MAR processing. These images are scanned at a tube potential of 80 kVp and a tube current of 50 mA (1.06 mGy), where images in rows 1 are window width/level (400/40) and rows 3 are images of window width/level (4024/1035). Rows 2 and 4 show the enlarged images of rows 1 and 3, respectively. Each row shows (**a**) non-metal (FBP), (**b**) non-MAR (FBP), (**c**) MAR (FBP), (**d**) non-metal (DLIR-H), (**e**) non-MAR (DLIR-H) and (**f**) MAR (DLIR-H) images in order.

Assessing and understanding the various metal artifacts that arise during the CT scan process is an important issue. There have been many attempts to identify metal artifacts seen in CT image and prevent them^2,24^. In addition, the development of the MAR technique to remove metal artifacts shown in the image is being studied continuously, and the recent deep learning-based MAR technology is gradually improving the performance of artifact reduction^25–27^. In this study, we confirmed the effect of removing metal artifacts by the MAR technique and the restoration performance of pixel information through the application of the MAR technique. Overall, the MAR technique restored the original image information well in the process of removing the metal artifacts, but it was confirmed that the restoration performance was poor in the structure with severe metal artifacts in very close region to the metal (Fig. 7). Unremoved metal artifacts and distortion by MAR technique not only affect diagnosis but can potentially cause misdiagnosis^12,16^. Therefore, it is necessary to understand not only the positive effects of the MAR technique, but also its limitations. We devised and used a new metric for full-reference based evaluation in this study, and the analysis area was extracted using automated segmentation algorithms without human subjective intervention. Because the MAR technique alters image quality, it is crucial to find the right balance between the MAR and the restoration of intrinsic pixel information. Our experimental results were important for understanding the effect on image quality due to the MAR technique.

This study used a phantom, not a human subject. The bones and tissues in phantom have a homogeneous structure, which is different from those of the human body. Since this study is a reference-based evaluation, it required an experimental procedure for insertion and removal of metal in the same measurement area. In addition, because structures such as tissues inside the human body are non-rigid, the shape and position of the measurement area may change depending on each CT scan. These are the reasons we conducted our experiments using the phantom. This method is not clinically applicable because our analysis method requires insertion and removal of metal. However, the ultimate purpose of this study is to understand the effect of MAR technique and its functional characteristics. This required experiments using controlled phantoms capable of quantitative analysis.

Our study has several limitations. First, the anatomic modeling method based on the pixel distribution characteristics used in this study has a limitation that “it cannot accurately distinguish the region corrected by MAR from the region distorted by MAR”. To distinguish between these two, selective segmentation of only the metal artifact region is required. If, ideally, the evaluation image (with metal) and the reference image (without metal) were scanned at exactly the same position without error in a perfectly homogeneous region, only the metal artifact region could be selectively extracted using the HU difference between the two images. However, even if the CT scan is performed as precisely as possible so that the position of the measurement target does not change, the difference between the two images will be unavoidable due to the vibrations caused by the CT table movement. We confirmed in our preliminary experiment that the phantom position was changed by the movement of the CT table in case of helical CT scan, which requires the movement of the CT table. So, we performed an axial CT scan to prevent the phantom movement due to the movement of the CT table, but the position change of the phantom that occurred in the process of replacing the metal by a person could not be avoided. In addition, it is not reasonable to judge the overall performance of MAR technique using the performance evaluation result by the MAR technique in a perfectly homogeneous object.

Second, this study was conducted with only one MAR technique of a specific manufacturer, and performance comparison with the MAR technique of other manufacturers was not performed. Therefore, the results drawn in this study are not representative of the performance of all existing MAR techniquese. Currently, the commercialized MAR technique is expected to have different levels of artifact reduction and tissue restoration for each manufacturer. We plan to compare various MAR features in follow-up studies.

## Supplementary Information

Supplementary Figure 1.

Supplementary Table 2.

## Data Availability

The datasets used and/or analyzed during the current study are available from the corresponding author upon reasonable request.
