# Supplementary material for: Comprehensive evaluation of artifact reduction and tissue recovery effects of metal artifact reduction technique based on full-reference metric
Source: Sci Rep. 2023 Jul 19;13:11679. doi: 10.1038/s41598-023-38516-9 (PMC10356954; doi:10.1038/s41598-023-38516-9)
Supplement: Supplementary file 1 — Supplementary Figure 1. [file 41598_2023_38516_MOESM1_ESM.docx]

**SUPPLEMENTARY FIGURE**

**
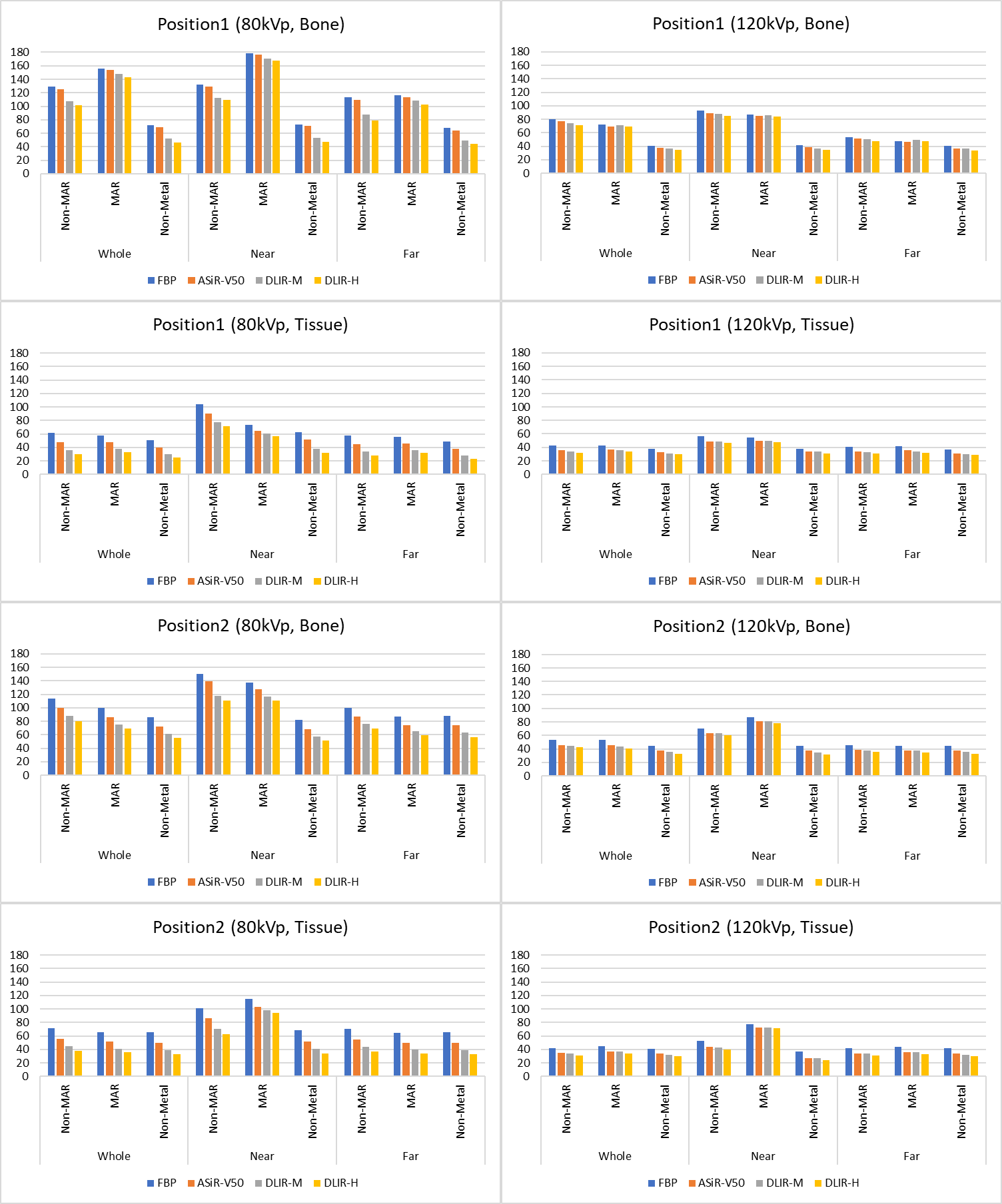
**

**Supplementary Figure 1.** Full width at half the maximum (FWHM) calculated from bone and tissue models

**
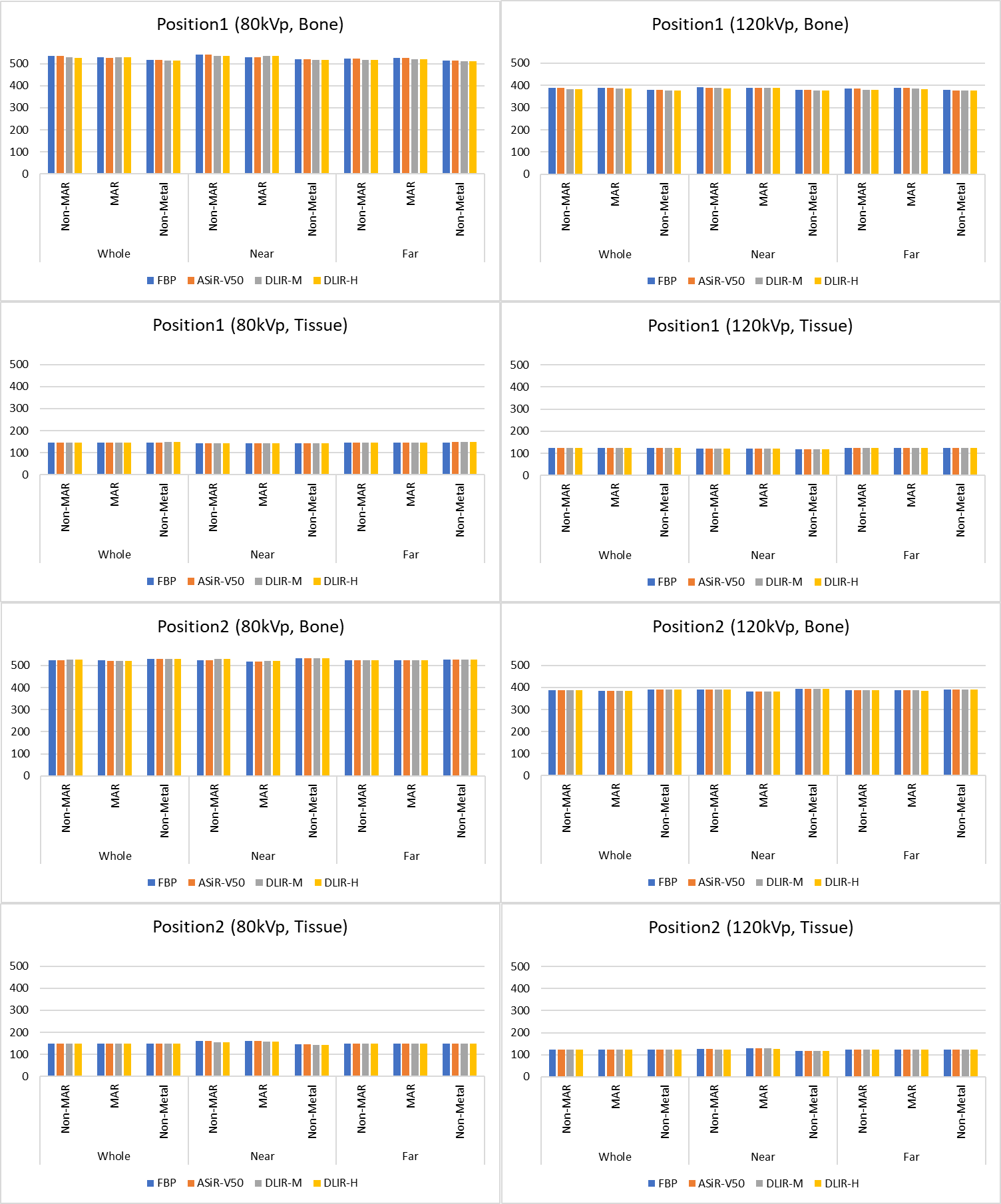
**

**Supplementary Figure 2.** Centroid calculated from bone and tissue models
