# Supplementary material for: Comprehensive evaluation of artifact reduction and tissue recovery effects of metal artifact reduction technique based on full-reference metric
Source: Sci Rep. 2023 Jul 19;13:11679. doi: 10.1038/s41598-023-38516-9 (PMC10356954; doi:10.1038/s41598-023-38516-9)
Supplement: Supplementary file 2 — Supplementary Table 2. [file 41598_2023_38516_MOESM2_ESM.docx]

**SUPPLEMENTARY TABLE**

**Supplementary table 1.** Full width at half the maximum (FWHM) calculated from bone and tissue models

| Region |  | Position1 (80kVp, 50mA^*^, Bone) | | | | Position1 (120kVp, 50mA^*^, Bone) | | | | |
| --- | --- | --- | --- | --- | --- | --- | --- | --- | --- | --- |
|  |  | FBP | ASiR-V50 | DLIR-M | DLIR-H | FBP | ASiR-V50 | DLIR-M | DLIR-H |  |
| Whole | Non-MAR | 129.19 | 125.57 | 107.69 | 101.64 | 80.04 | 76.89 | 74.51 | 71.73 |  |
|  | MAR | 155.68 | 153.94 | 147.83 | 143.24 | 72.03 | 69.66 | 71.22 | 69.26 |  |
|  | Non-Metal | 71.43 | 68.88 | 51.84 | 45.87 | 41.13 | 38.10 | 36.60 | 34.32 |  |
| Near | Non-MAR | 132.41 | 129.27 | 112.77 | 109.05 | 93.04 | 89.26 | 87.61 | 85.15 |  |
|  | MAR | 178.43 | 176.48 | 170.41 | 167.16 | 87.38 | 84.75 | 85.71 | 84.08 |  |
|  | Non-Metal | 72.68 | 70.54 | 53.08 | 46.74 | 41.44 | 38.63 | 36.77 | 34.68 |  |
| Far | Non-MAR | 113.62 | 109.24 | 87.49 | 78.92 | 53.28 | 51.23 | 50.38 | 47.42 |  |
|  | MAR | 116.28 | 113.69 | 108.53 | 102.26 | 47.91 | 46.58 | 49.91 | 47.54 |  |
|  | Non-Metal | 68.12 | 64.23 | 48.70 | 43.78 | 40.64 | 37.12 | 36.40 | 33.66 |  |
| Region |  | Position1 (80kVp, 50mA, Tissue) | | | | Position1 (120kVp, 50mA, Tissue) | | | | |
|  |  | FBP | ASiR-V50 | DLIR-M | DLIR-H | FBP | ASiR-V50 | DLIR-M | DLIR-H |  |
| Whole | Non-MAR | 61.78 | 47.92 | 36.03 | 29.62 | 42.28 | 35.45 | 34.21 | 31.80 |  |
|  | MAR | 57.30 | 47.35 | 37.99 | 33.40 | 43.06 | 37.02 | 35.73 | 33.45 |  |
|  | Non-Metal | 50.73 | 40.01 | 29.68 | 24.69 | 37.29 | 32.34 | 31.28 | 29.62 |  |
| Near | Non-MAR | 103.79 | 89.95 | 77.28 | 71.03 | 56.61 | 49.06 | 49.03 | 46.18 |  |
|  | MAR | 73.74 | 64.16 | 60.43 | 56.21 | 54.31 | 49.98 | 49.54 | 47.44 |  |
|  | Non-Metal | 63.03 | 52.20 | 38.18 | 31.95 | 38.02 | 33.82 | 33.49 | 31.22 |  |
| Far | Non-MAR | 58.02 | 44.84 | 33.48 | 27.53 | 40.75 | 34.01 | 32.82 | 30.45 |  |
|  | MAR | 55.33 | 45.36 | 35.88 | 31.48 | 41.69 | 35.53 | 34.27 | 32.00 |  |
|  | Non-Metal | 48.83 | 38.09 | 27.86 | 22.98 | 36.47 | 31.32 | 30.08 | 28.39 |  |
| Region |  | Position2 (80kVp, 50mA, Bone) | | | | Position2 (120kVp, 50mA, Bone) | | | |  |
|  |  | FBP | ASiR-V50 | DLIR-M | DLIR-H | FBP | ASiR-V50 | DLIR-M | DLIR-H |  |
| Whole | Non-MAR | 113.54 | 99.97 | 87.82 | 80.20 | 52.95 | 45.56 | 44.93 | 42.10 |  |
|  | MAR | 99.39 | 85.76 | 75.54 | 69.06 | 53.03 | 45.04 | 43.51 | 40.21 |  |
|  | Non-Metal | 85.99 | 72.40 | 61.65 | 55.12 | 44.68 | 37.99 | 35.83 | 33.02 |  |
| Near | Non-MAR | 149.78 | 139.78 | 117.73 | 110.43 | 69.67 | 63.68 | 63.68 | 60.51 |  |
|  | MAR | 137.00 | 127.09 | 117.03 | 111.23 | 86.77 | 81.14 | 80.49 | 77.99 |  |
|  | Non-Metal | 81.97 | 68.55 | 57.83 | 51.52 | 44.09 | 37.14 | 34.62 | 32.04 |  |
| Far | Non-MAR | 100.15 | 86.71 | 76.49 | 69.50 | 45.83 | 38.65 | 37.84 | 35.41 |  |
|  | MAR | 87.05 | 73.98 | 65.28 | 59.62 | 44.71 | 37.97 | 37.35 | 34.58 |  |
|  | Non-Metal | 88.09 | 74.19 | 62.95 | 56.2 | 44.24 | 37.56 | 35.64 | 32.62 |  |
| Region |  | Position2 (80kVp, 50mA, Tissue) | | | | Position2 (120kVp, 50mA, Tissue) | | | |  |
|  |  | FBP | ASiR-V50 | DLIR-M | DLIR-H | FBP | ASiR-V50 | DLIR-M | DLIR-H |  |
| Whole | Non-MAR | 71.28 | 55.47 | 44.74 | 38.10 | 42.13 | 34.81 | 33.93 | 31.52 |  |
|  | MAR | 65.95 | 51.39 | 41.37 | 35.52 | 44.50 | 37.29 | 36.59 | 34.11 |  |
|  | Non-Metal | 65.87 | 49.93 | 39.22 | 32.80 | 41.42 | 33.65 | 32.42 | 30.03 |  |
| Near | Non-MAR | 100.66 | 86.65 | 70.15 | 62.36 | 52.36 | 44.14 | 42.42 | 39.48 |  |
|  | MAR | 114.61 | 103.23 | 97.76 | 93.80 | 77.77 | 72.36 | 72.82 | 71.18 |  |
|  | Non-Metal | 68.76 | 51.71 | 40.66 | 34.01 | 36.78 | 27.07 | 27.29 | 24.31 |  |
| Far | Non-MAR | 70.21 | 54.42 | 43.84 | 37.29 | 41.66 | 34.38 | 33.56 | 31.18 |  |
|  | MAR | 64.46 | 50.05 | 40.14 | 34.45 | 43.48 | 36.36 | 35.70 | 33.23 |  |
|  | Non-Metal | 65.67 | 49.76 | 39.01 | 32.58 | 41.55 | 33.88 | 32.49 | 30.12 |  |

Note.-FWHM calculated from bone and tissue models. FWHM = full width at half the maximum, MAR = metal artifact reduction, FBP = filtered back-projection, ASiR-V = adaptive statistical iterative reconstruction veo, DLIR = deep learning-based image reconstruction (Medium, High)

^*^CTDIvol values were 1.06 and 3.15mGy for (80kVp, 50mA) and (120kVp, 50mA), respectively.

**Supplementary table 2.** Centroid calculated from bone and tissue models

| Region |  | Position1 (80kVp, 50mA, Bone) | | | | Position1 (120kVp, 50mA, Bone) | | | | |
| --- | --- | --- | --- | --- | --- | --- | --- | --- | --- | --- |
|  |  | FBP | ASiR-V50 | DLIR-M | DLIR-H | FBP | ASiR-V50 | DLIR-M | DLIR-H |  |
| Whole | Non-MAR | 535.17 | 534.43 | 528.39 | 527.08 | 387.99 | 387.56 | 384.15 | 383.73 |  |
|  | MAR | 527.51 | 526.96 | 529.26 | 528.81 | 388.99 | 388.97 | 386.02 | 385.91 |  |
|  | Non-Metal | 517.42 | 517.25 | 514.20 | 513.79 | 378.89 | 378.46 | 376.19 | 375.91 |  |
| Near | Non-MAR | 541.36 | 540.39 | 534.65 | 533.53 | 390.61 | 389.89 | 387.25 | 387.01 |  |
|  | MAR | 529.77 | 528.9 | 535.20 | 535.27 | 389.39 | 388.98 | 387.22 | 387.44 |  |
|  | Non-Metal | 518.74 | 518.55 | 515.94 | 515.49 | 379.02 | 378.68 | 376.44 | 376.20 |  |
| Far | Non-MAR | 521.99 | 521.86 | 515.99 | 515.42 | 385.04 | 384.75 | 380.47 | 379.87 |  |
|  | MAR | 526.08 | 525.98 | 521.14 | 520.08 | 389.19 | 389.76 | 384.70 | 384.14 |  |
|  | Non-Metal | 514.59 | 514.56 | 510.69 | 510.31 | 378.61 | 378.02 | 375.63 | 375.30 |  |
| Region |  | Position1 (80kVp, 50mA, Tissue) | | | | Position1 (120kVp, 50mA, Tissue) | | | | |
|  |  | FBP | ASiR-V50 | DLIR-M | DLIR-H | FBP | ASiR-V50 | DLIR-M | DLIR-H |  |
| Whole | Non-MAR | 145.22 | 145.95 | 146.56 | 146.85 | 123.13 | 123.43 | 123.70 | 123.83 |  |
|  | MAR | 145.24 | 145.76 | 146.16 | 146.41 | 123.16 | 123.32 | 123.20 | 123.26 |  |
|  | Non-Metal | 146.50 | 147.15 | 147.43 | 147.79 | 123.49 | 123.66 | 123.99 | 124.10 |  |
| Near | Non-MAR | 142.69 | 142.71 | 141.79 | 141.65 | 120.85 | 120.84 | 120.49 | 120.65 |  |
|  | MAR | 143.57 | 143.46 | 143.14 | 142.93 | 120.50 | 120.60 | 119.80 | 119.78 |  |
|  | Non-Metal | 142.87 | 142.52 | 141.35 | 141.65 | 118.38 | 118.41 | 117.77 | 117.78 |  |
| Far | Non-MAR | 145.56 | 146.27 | 146.79 | 147.04 | 123.37 | 123.70 | 123.97 | 124.09 |  |
|  | MAR | 145.48 | 146.04 | 146.45 | 146.72 | 123.50 | 123.63 | 123.54 | 123.58 |  |
|  | Non-Metal | 147.00 | 147.73 | 148.13 | 148.46 | 124.33 | 124.49 | 124.93 | 125.08 |  |
| Region |  | Position2 (80kVp, 50mA, Bone) | | | | Position2 (120kVp, 50mA, Bone) | | | |  |
|  |  | FBP | ASiR-V50 | DLIR-M | DLIR-H | FBP | ASiR-V50 | DLIR-M | DLIR-H |  |
| Whole | Non-MAR | 524.49 | 523.80 | 525.46 | 525.68 | 388.85 | 388.54 | 387.97 | 388.12 |  |
|  | MAR | 522.62 | 521.61 | 521.85 | 521.94 | 385.66 | 385.43 | 384.87 | 384.83 |  |
|  | Non-Metal | 530.19 | 529.82 | 529.68 | 529.56 | 391.81 | 391.68 | 391.46 | 391.56 |  |
| Near | Non-MAR | 523.72 | 523.23 | 528.63 | 528.86 | 391.79 | 391.67 | 391.60 | 391.79 |  |
|  | MAR | 518.71 | 517.85 | 520.01 | 520.20 | 381.78 | 381.24 | 381.34 | 381.27 |  |
|  | Non-Metal | 533.30 | 533.10 | 533.59 | 533.49 | 394.73 | 394.56 | 394.55 | 394.76 |  |
| Far | Non-MAR | 524.83 | 524.04 | 524.41 | 524.68 | 387.69 | 387.46 | 386.80 | 386.96 |  |
|  | MAR | 524.40 | 523.49 | 522.95 | 523.00 | 386.96 | 386.65 | 385.99 | 385.94 |  |
|  | Non-Metal | 528.07 | 527.53 | 526.90 | 526.73 | 389.87 | 389.73 | 389.30 | 389.35 |  |
| Region |  | Position2 (80kVp, 50mA, Tissue) | | | | Position2 (120kVp, 50mA, Tissue) | | | |  |
|  |  | FBP | ASiR-V50 | DLIR-M | DLIR-H | FBP | ASiR-V50 | DLIR-M | DLIR-H |  |
| Whole | Non-MAR | 148.52 | 148.49 | 148.66 | 148.81 | 124.15 | 124.28 | 124.58 | 124.59 |  |
|  | MAR | 148.23 | 148.35 | 148.76 | 148.88 | 123.60 | 123.78 | 124.11 | 124.11 |  |
|  | Non-Metal | 147.90 | 148.01 | 148.46 | 148.66 | 123.24 | 123.23 | 123.51 | 123.51 |  |
| Near | Non-MAR | 159.85 | 159.74 | 154.67 | 154.68 | 125.36 | 124.83 | 123.89 | 123.57 |  |
|  | MAR | 160.64 | 159.91 | 157.66 | 157.32 | 130.50 | 128.92 | 128.17 | 127.18 |  |
|  | Non-Metal | 145.67 | 144.55 | 143.87 | 144.03 | 118.50 | 118.30 | 117.91 | 117.99 |  |
| Far | Non-MAR | 148.20 | 148.21 | 148.51 | 148.67 | 124.09 | 124.26 | 124.60 | 124.62 |  |
|  | MAR | 148.08 | 148.28 | 148.76 | 148.88 | 123.49 | 123.74 | 124.09 | 124.12 |  |
|  | Non-Metal | 148.03 | 148.21 | 148.72 | 148.93 | 123.56 | 123.62 | 123.93 | 123.95 |  |

Note.-Centroid calculated from bone and tissue models. FWHM = full width at half the maximum, MAR = metal artifact reduction, FBP = filtered back-projection, ASiR-V = adaptive statistical iterative reconstruction veo, DLIR = deep learning-based image reconstruction (Medium, High)

^*^CTDIvol values were 1.06 and 3.15mGy for (80kVp, 50mA) and (120kVp, 50mA), respectively.
